# Immunological Responses to Cancer Therapy

**DOI:** 10.3390/ijms23136989

**Published:** 2022-06-23

**Authors:** Marieke F. Fransen

**Affiliations:** Department of Pulmonary Medicine, Cancer Center Amsterdam, Amsterdam Institute for Infection and Immunity, Amsterdam UMC, De Boelelaan 1117, 1081 HV Amsterdam, The Netherlands; m.f.fransen@amsterdamumc.nl

The use of immunotherapy for cancer has taken flight in the last two decades, from experimental therapy envisioned mainly by laboratory scientists to everyday treatment used by physicians to treat many patients. Since the great success of FDA approval of ipilimumab for stage IV melanoma patients [1], the usage of checkpoint-blocking antibody and other forms of immunotherapy for cancer has grown exponentially. After the introduction of immunotherapy as a standard therapy modality in the repertoire of cancer treatment, it is time to take a step back and evaluate. In order to implement and exploit the potential of immunotherapy modalities to their fullest, we need to make intelligent choices about combinatorial treatments and fill the caveats of knowledge that currently exist.

Immunotherapy for cancer requires a different way of thinking compared to other types of cancer treatment. Whereas chemotherapy and radiotherapy are aimed entirely at killing malignant cells and possibly any other healthy bystander cells, immunotherapy targets immune cells and activates them, upon which they can very specifically kill malignant cells without harming bystander cells. This revolutionary therapy requires a different approach to side-effects, combinatorial treatments, dosing, and timing schedules.

In this issue, several aspects of cancer therapy and immunotherapy are investigated that warrant further exploration. By investigating underappreciated areas of the immunology surrounding cancer and treatment, we aim to fill some of the knowledge gaps that presently exist.

The most used immunotherapy at present is checkpoint blockade. Although widely implemented in clinical practice, there is still room for improvement, both in clinical efficacy and in practical use and knowledge. Several manuscripts in this issue are therefore devoted to aspects regarding checkpoint therapy. Bareke et al. [2] describe, in their review, the current knowledge on autoimmunity and side-effects of immunotherapy, thereby highlighting an immunotherapy-specific problem that is a real threat to clinical implementation of checkpoint blockades. Kulikowska de Nalecz et al. [3] and An et al. [4] publish on biomarkers in blood, an easily accessible source. These publications are aimed at making stratification of patients possible in order to avoid treating patients with a low chance of treatment efficacy, or a high chance of dangerous side effects.

Both Lisini et al. [5] and Fransen et al. [6] describe the possibilities, advantages, and disadvantages of local administration of immunotherapy, the first in the context of mesothelioma, an aggressive form of lung cancer that might benefit from more localized treatment, and the latter in the context of targeting the tumor-draining lymph nodes, highlighting the pivotal role of this immune orchestrating organ. These novel approaches warrant more appreciation, since they target the main areas, tumor and tumor-draining lymph nodes, reduce possible side-effects, and, most importantly, lead to systemic immune responses. Investigating the tumor-draining lymph node is of vital importance in taking immunotherapy to the next level, since this organ has recently been proven to be crucially important for the efficacy of immunotherapy [7,8,9,10].

In three other manuscripts in this issue, under-evaluated aspects of checkpoint therapy are explored. Both Muller et al. [11] and Morais Melo et al. [12] describe novel targets in cancer that might lead to antigens and T cell activation, namely endogenous retroviruses and somatic mutations. Additionally, Gavali et al. [13] describe molecular aspects of T cell activation in cancer immunotherapy, focusing on the role of ubiquitination. Studying these aspects will lead to improved understanding and possibly increased implementation of checkpoint therapy, which is, so far, is regarded as a treatment for tumors with a high mutational burden.

Another form of successful immunotherapy of cancer that has been implemented in the clinic is Car-T cell therapy. Two manuscripts in this issue describe Car-T cell therapy; namely, Martinez-Rubio et al. [14] describe a mathematical model to evaluate dynamics of Car-T cells in bone marrow, and Pietrobon et al. [15] review current literature on the persistence of Car-T cells. Both manuscripts add to the current effort to further improve this already very successful therapy modality.

In this issue of the *International Journal of Molecular Science*, several under-evaluated aspects regarding immunological responses to cancer are described. Knowledge gained from this will aid the implementation of new combinatorial strategies and more efficient treatment designs of immunotherapy.
